# Ultra-structural mapping of sugarcane bagasse after oxalic acid fiber expansion (OAFEX) and ethanol production by *Candida shehatae* and *Saccharomyces cerevisiae*

**DOI:** 10.1186/1754-6834-6-4

**Published:** 2013-01-16

**Authors:** Anuj K Chandel, Felipe F A Antunes, Virgilio Anjos, Maria J V Bell, Leonarde N Rodrigues, Om V Singh, Carlos A Rosa, Fernando C Pagnocca, Silvio S da Silva

**Affiliations:** 1 Department of Biotechnology, University of São Paulo, School of Engineering of Lorena, Estrada Municipal do Campinho- Caixa,, Postal 116 12.602.810, Lorena/SP, Brazil; 2 Material Spectroscopy Laboratory, Department of Physics, Federal University of Juiz de Fora,, 36036-330, Juiz de Fora, MG, Brazil; 3 Division of Biological and Health Sciences, University of Pittsburgh, 16701, Bradford, PA, USA; 4 Department of Microbiology, Federal University of Minas Gerais,, Belo Horizonte, MG, Brazil; 5 Department of Biochemistry and Microbiology, Institute of Biosciences CEIS/UNESP – Rio, Claro/ SP, Brazil

## Abstract

**Background:**

Diminishing supplies of fossil fuels and oil spills are rousing to explore the alternative sources of energy that can be produced from non-food/feed-based substrates. Due to its abundance, sugarcane bagasse (SB) could be a model substrate for the second-generation biofuel cellulosic ethanol. However, the efficient bioconversion of SB remains a challenge for the commercial production of cellulosic ethanol. We hypothesized that oxalic-acid-mediated thermochemical pretreatment (OAFEX) would overcome the native recalcitrance of SB by enhancing the cellulase amenability toward the embedded cellulosic microfibrils.

**Results:**

OAFEX treatment revealed the solubilization of hemicellulose releasing sugars (12.56 g/l xylose and 1.85 g/l glucose), leaving cellulignin in an accessible form for enzymatic hydrolysis. The highest hydrolytic efficiency (66.51%) of cellulignin was achieved by enzymatic hydrolysis (Celluclast 1.5 L and Novozym 188). The ultrastructure characterization of SB using scanning electron microscopy (SEM), atomic force microscopy (AFM), Raman spectroscopy, Fourier transform–near infrared spectroscopy (FT-NIR), Fourier transform infrared spectroscopy (FTIR), and X-ray diffraction (XRD) revealed structural differences before and after OAFEX treatment with enzymatic hydrolysis. Furthermore, fermentation mediated by *C. shehatae* UFMG HM52.2 and *S. cerevisiae* 174 showed fuel ethanol production from detoxified acid (3.2 g/l, yield 0.353 g/g; 0.52 g/l, yield, 0.246 g/g) and enzymatic hydrolysates (4.83 g/l, yield, 0.28 g/g; 6.6 g/l, yield 0.46 g/g).

**Conclusions:**

OAFEX treatment revealed marked hemicellulose degradation, improving the cellulases’ ability to access the cellulignin and release fermentable sugars from the pretreated substrate. The ultrastructure of SB after OAFEX and enzymatic hydrolysis of cellulignin established thorough insights at the molecular level.

## Background

Multidimensional applications have increased the demand for fossil fuels, sending disturbing signals regarding low levels of crude oil underground and the burden on aging refineries [1]. Recent developments have explored marginally meaningful alternatives to fossil fuels such as bioethanol using corn grains or sugarcane juice as substrates, which would result in tremendous price hikes for basic food commodities around the world [2]. However, bioethanol produced from sustainable feedstocks such as lignocellulosics has drawn worldwide attention as a legitimate alternative to gasoline. The development of green gasoline or ethanol from abundantly available cellulosic materials in nature is gaining significant momentum as a sustainable mitigation strategy [3]. As an alternative to fossil and food-based fuels, cellulosic ethanol offers near-term environmental sustainability benefits. However, the selection of a raw substrate and its efficient utilization are critical steps in the process of economization. Brazil and India are the largest producers of sugarcane, with an annual production of about 650 and 350 MMT/year respectively [4], able to supply sugarcane bagasse (SB) in abundance round the year.

SB consists of crystalline cellulose nanofibrils embedded in an amorphous matrix of cross-linked lignin and hemicelluloses that impairs enzyme and microbial accessibility [5]. Structural changes in the cellular components of SB have been studied after pretreatment with formic acid [6], sono-assisted acid hydrolysis [7], and sequential acid–base [8] methodologies. However, visual characterizations of cellular components elucidating the hemicellulose degradation and delocalization of lignin after oxalic acid fiber expansion (OAFEX) pretreatment have yet to be performed. Cell wall anatomy of SB and macroscopic/microscopic barriers for cellulase-mediated saccharification reveal that cell wall hydrolysis encompasses several orders of magnitude (10^0^-10^-9^ meters [9,10].

Oxalic acid (OA) is a strong dicarboxylic acid with higher catalytic efficiency than the sulfuric acid, and acts primarily upon hemicellulose. Pretreatment with OA leaves cellulignin in a fragile form, making it amenable to concerted cellulolytic enzyme action on cellulose, which yields glucose. The current pretreatment methodologies used to degrade the holocellulosic (cellulose + hemicellulose) fraction of the plant cell wall have economic and environmental limitations. However, OAFEX has been found to be an efficient pretreatment strategy for hemicellulose removal from giant reed [11], *Saccharum spontaneum*[12], corn-cobs [13], and wood chips [14]. Therefore, a comprehensive structural analysis of the nanoscale architecture of OAFEX-treated cell walls in tandem with molecular changes will assist to explore the fundamental mechanisms of biomass recalcitrance.

The fermentation of pentose and hexose sugars present in lignocellulose hydrolysate is important to produce “economic ethanol” [15,16]. *Candida shehatae*, native xylose-fermenting yeast, has shown the capability to utilize pentose sugars efficiently for ethanol production [17,18]. The microorganism *Saccharomyces cerevisiae* is a perennial choice for bioethanol production from glucose-rich cellulosic hydrolysates [19]. We attempted to pretreat SB using OA followed by enzymatic hydrolysis. The released sugars (12.56 g/l xylose and 1.85 g/l glucose) were than subjected to ethanol fermentation using *C. shehatae* UFMG HM 52.2 and *S. cerevisiae* 174 in batch fermentation. A multiscale structural analysis of native, OAFEX-treated and enzyme-digested SB was performed using scanning electron microscopy (SEM), atomic force microscopy (AFM), X-ray diffraction (XRD), Raman spectroscopy, near infrared spectroscopy (NIR), and Fourier transform infrared (FTIR) analysis, and revealed the structural differences before and after OAFEX treatment and enzymatic hydrolysis.

## Results and discussion

The abundance availability of SB as substrate can be used for fuel ethanol production without jeopardizing food and feed production. SB is rich in lignocellulosics contained (% d. wt.): cellulose 45.0, hemicellulose 25.8, lignin 19.1, structural ash 1.0, extractive 9.1 [20]. The holocellulosic (hemicellulose + cellulose) content of SB (*circa* 70%) is fairly comparable with other lignocellulosic materials studied for ethanol production, such as wheat straw (54%), birch (73%), spruce (63.2%) (11), corn stover (59.9%), and poplar (58.2%) [21]. Biomass recalcitrance and efficient sugar conversion into ethanol are among the key hindrances to ethanol production that are preventing biorefineries from taking a central role in the energy sector.

### OAFEX treatment of sugarcane bagasse and detoxification of hemicellulosic hydrolysate

OA is a strong organic acid, and due to its di-carboxylic properties, is better for hemicellulose hydrolysis than mineral acids such as sulfuric or hydrochloric acid [13]. OAFEX pretreatment is known for its precise action to degrade hemicellulose with fewer inhibitors and increased surface area of cellulose for improved enzymatic action [11,13,14,22,23]. The dilute OA hydrolysis of SB (160°C; 3.5% OA w/v; 10% total solids; 20 min residence time) depolymerized the hemicellulose fraction of the bagasse cell wall into sugars (xylose 12.56 g/l, glucose 1.85 g/l, arabinose 0.85 g/l) with the hemicellulose conversion (90.45%) and inhibitors (0.234 g/l furfurals, 0.103 g/l 5-hydroxy methyl furfural (5-HMF), 1.47 g/l acetic acid and 2.95 g/l total phenolics) (Table  1). Diluted OA hydrolysis degrades the hemicellulosic fraction of the plant cell wall into its monomeric constituents, such as xylose and other sugars, in addition to inhibitory compounds [13,22].

**Table 1:** Sugarcane bagasse hemicellulosic hydrolysate profile after detoxification by calcium hydroxide overliming

**Constituents**	**Quantity (g/l)**	**% Reduction after overliming**	**Residual constituents quantity (g/l)**
Xylose	12.56	7.51	11.61
Glucose	1.85	25.50	1.37
Arabinose	0.85	8.56	0.777
Acetic acid	1.47	5.54	1.38
Furfural	0.242	23.96	0.184
5-HMF	0.094	10.63	0.084
Total phenolics	2.95	45.5	1.60

The values are mean of three replicates. Standard deviation was within 10%.

After hemicellulose is removed from the substrate during the OAFEX treatment, the leftover solid material, called cellulignin, becomes accessible for enzymatic saccharification. Critical factors such as lignocellulosic substrate, temperature, acid load, residence time, and substrate-to-liquid ratio play key roles in breaking down hemicellulose into its monomeric constituents [13,14], in addition to releasing inhibitors from the substrate [17]. Scordia et al. [12] reported 30.70 g/L xylose (93.73% conversion), 2.60 g/L glucose (54.17% conversion), 1.40 g/L arabinose, 3.60 g/L acetic acid, 0.68 g/L furfurals, 0.10 g/L HMF, and 6.58 g/L phenolics from *S. spontaneum* under the hydrolytic conditions (158°C, 16 min, 3.21% w/w OA, solid-to-liquid ratio of 1:4). The OA-mediated hydrolysis of giant reed (*Arundo donax* L.) revealed 100% recovery of xylose, arabinose, glucose, 6.55 g/L furfural, and 0.18 g/L HMF at defined hydrolysis conditions, *i.e.* 190°C, 25 min, 5% w/w OA [11]. Maize residues pretreated with OA (160°C, 1.8% OA, 10 min residential time) showed 28±2.5% w/w xylose yield [23]. These studies reveal the potential of OA pretreatment for hemicellulose degradation into fermentable sugars.

Fermentative inhibitors are common in acidic hydrolysis of SB. OA hydrolysate can be detoxified effectively using calcium hydroxide overliming: raising the pH of hydrolysate to 10.0. Regardless the source of availability of lignocellulosic hydrolysate, the detoxification using calcium hydroxide overliming is tedious and a subject of further investigation. It is an intensive step which cause precipitation and stirring problems. The re-adjustment to pH 6.0 showed efficient removal of inhibitors from the hydrolysate (Table  1). After Ca(OH)_2_ overliming of the SB hemicellulosic hydrolysate, a significant reduction in furfurals (23.96%), 5-HMF (10.63%), total phenolics (45.5%), and acetic acid (5.54%) was observed with a marginal loss in xylose (7.51%), glucose (25.50%), and arabinose (8.56%) (Table 1). During Ca(OH)_2_ overliming at pH 10, precipitation of furfurals and phenolics occurs, resulting in their removal during vacuum filtration of the hydrolysate. Earlier, we have reported 41.75% and 33.21% reductions in furans and phenolics from *S. spontaneum* hemicellulosic hydrolysates after Ca(OH)_2_ overliming [24]. There was only a 5.5% reduction in acetic acid content after overliming. In another study, Rodrigues et al. [22] observed no change in acetic acid concentration after overliming of corn stover hemicellulosic hydrolysate.

### Enzymatic hydrolysis

**Figure 1:**
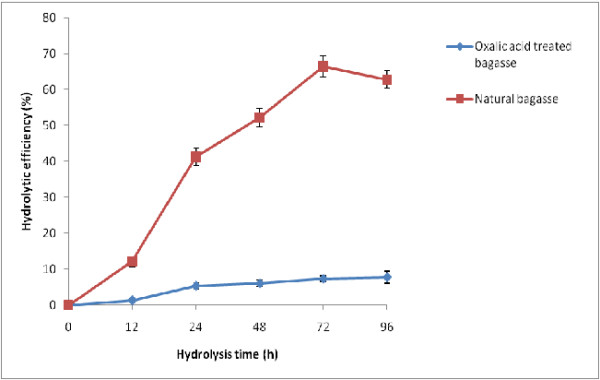
**Enzymatic hydrolysis profile of oxalic acid pretreated sugarcane bagasse.** The values are mean of three replicates. Standard deviation was within 10%.

OAFEX-treated bagasse was enzymatically hydrolyzed with commercial enzymes, i.e., 20 FPU/g of Celluclast 1.5 L and 30 IU/g of Novozym 188 at 50°C for 96 h in the presence of tween-20. Enzymatic hydrolysis after 72 h revealed 28.5 g/L glucose release with 66.51% saccharification efficiency (Figure  1). The OAFEX-treated bagasse showed a concomitant increase in sugar recovery up to 72 h followed by a decrease in glucose concentration at 96 h of incubation. These enzyme loadings in the presence of surfactants are sufficient to hydrolyse the cellulose present in pretreated substrate.

A similar trend was reported in the sugar recovery during enzymatic hydrolysis of aqueous-ammonia-pretreated *S. spontaneum*[25]. The amounts of enzymes required for hydrolysis of pretreated raw material depend upon the pretreatment applied to the substrate and the availability of carbohydrate content in the substrate [26]. A maximum yield of sugars (482±22 mg/g, 64% hydrolytic efficiency) from acidic-hydrolyzed wheat straw (7.83% DS, acid loading 0.75%) was obtained after enzymatic hydrolysis [26]. We also observed 28.5 g/l glucose representing 66.51% efficiency after enzymatic hydrolysis of OAFEX-treated bagasse in the presence of tween-20 (2.5 g/l). Zheng et al. [27] observed that high enzyme loadings did not alter saccharification and yields. Rezende et al. [8] reported 72% cellulose conversion from consecutive acid–base pretreated SB. A 65% cellulose conversion was obtained after the enzymatic hydrolysis (1.91% w/w pretreated SB, 20 FPU/g enzyme loading, 0.05 g/g surfactant) of bagasse pretreated with dilute sulfuric acid (1.75% w/w bagasse content, 1.7% w/w H_2_SO_4_ loading, 150°C, and 30 min pretreatment time) [20]. Our results indicate that hemicellulose removal and the possible relocalization of lignin moieties during OAFEX treatment could yield the desired amount of sugar toward the goal of developing an intensified and simplified process for cellulose saccharification.

### Ultra structural characterization of native and treated SB

SB, the biomass is naturally built through a special arrangement of cross-linked lignin with a holocellulose network, providing a superb mechanism to protect itself from microbial invasions in nature [5]. Rendering the carbohydrate fraction of the cell wall accessible is a multiscale phenomenon encompassing several orders of magnitude due to both macroscopic (compositional heterogeneity, mass transfer limitations) and microscopic barriers (holocellulose crystallinity, lignin-cellulose linkage) [28]. The pretreatment allows cellular breakdown, increasing the amenability of enzymes for sugar monomer recovery. The SEM and AFM of cell walls after acidic treatment reveal disorganization of cell wall components, which is pivotal for improved cellulase action on the carbohydrate polymer in order to yield simple sugars.

### Cell wall composition

A physico-chemical analysis of the native SB cell wall revealed a composition (dry weight) of 24.67% total lignin (20.88% Klason lignin + 3.79% acid soluble lignin), 41.22% cellulose, 25.62% hemicellulose, 2.90% extractives, 8.16% moisture, and 1.5% structural ash. This composition of SB is in agreement with earlier studies [20]. The treated substrate after OA pretreatment of SB revealed 58.84% cellulose, 8.75% hemicellulose, and 24.56% total lignin (24.18% Klason lignin + 0.38% acid soluble lignin). The total lignin content in natural and OA-pretreated bagasse was determined to be almost similar (OA: 24.56%; natural: 24.67%). Klason lignin content increased in the OA-treated bagasse (24.18%) compared to the native bagasse (20.88%). However, acid-soluble lignin was removed maximally in the OA-pretreated bagasse, making the total content almost similar.

The pretreatment of corn cob with OA showed 100% removal of hemicellulose with a partial increase in lignin. Lee et al. [13] observed 6.76% xylan, 35.09% glucan, 0.12% galactan, 0.55% arabinan, and 12.09% lignin in OA-pretreated corn cob (168°C, 26 min, 30 g/l OA, 1:6 solid-to-liquid ratio) compared with the native material (27.86% xylan, 37.07% glucan, 0.61% galactan, 2.19% arabinan, and 13.92% lignin). The changes in cell wall composition of lignocellulosic material after OA pretreatment depend upon the nature of the substrate and the conditions explored during pretreatment [11-13].

### Scanning electron microscopy (SEM)

**Figure 2:**
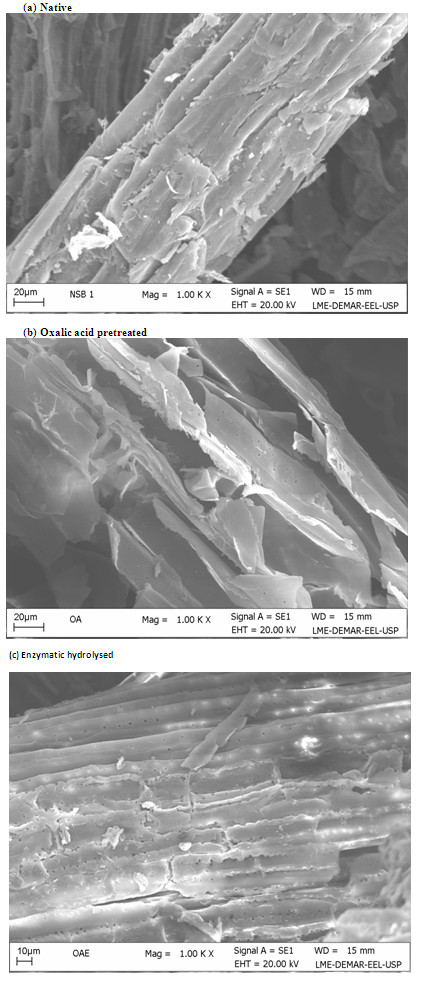
**Scanning electron microscopy surface images of the sugarcane bagasse. (a)** Native **(b)** Oxalic acid pretreated **(c)** Enzymatic hydrolysed. Compacted surface of cell wall showing waxes and deposits in native SB, disruption of cell wall was more evident after oxalic acid mediated pretreatment and enzymatic hydrolysis.

SEM analysis indicated the cell wall degradation, and surface properties of native, OA-pretreated, and enzymatically digested SB (Figure  2a-c). The native SB cell wall showed parallel stripes and waxes, extractives, and other deposits on the surface (Figure  2a). Obtained results are in agreement with earlier studies, where OA was reported to disrupt the structure of fibers and pith by removing the hemicellulose fraction of the cell wall, waxes, and other deposits. OAFEX leaves the overall structure disorganized, simultaneously increasing the surface area for enzymatic action [14,23].

OA-pretreated bagasse fiber showed small pores on the surface and fiber disruption, which revealed the efficacy of the pretreatment process (Figure  2b). The OAFEX-treated SB revealed disruption of cell wall after enzymatic hydrolysis. Similarly, Rezende et al. [8] observed a disrupted fiber surface and exposed parallel stripes after sulfuric acid pretreatment of SB. Our data is in agreement and indicates severe disruption and exposure of parallel stripes in OAFEX-treated SB (Figure  2b). After the enzyme treatment of SB, the analysis showed maximum disintegration and numerous holes in the cell wall, verifying the enzymatic action on cellulose (Figure  2c). The exposure of cellulose through structural alteration of the bagasse is the crucial factor in hydrolysis of the remaining cellulosic fraction present in the cell wall. Similar observations have been reported from enzymatic hydrolysis of dilute-sulfuric-acid-pretreated *S. spontaneum*[24,25]. Kristensen et al. [29] also observed similar effect on wheat straw cell walls after hydrothermal pretreatment.

### Atomic force microscopy (AFM)

**Figure 3:**
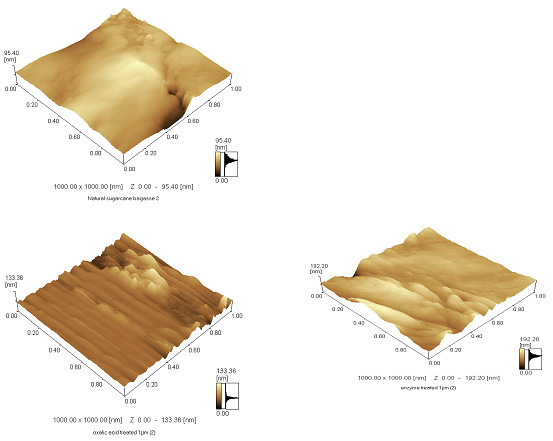
**Atomic force microscopy (AFM) amplitude images. (a)**Native sugarcane bagasse **(b)** Oxalic acid-pretreated bagasse **(c)** Enzyme hydrolysed bagasse. AFM scan revealed the cross linked cellulose + hemicellulose network in native SB. After OA pretreatment, non homogenous surface appeared with globular surface deposition (characteristic of lignin). AFM tip indicated affinity towards hydrophilic areas (192 nm) revealing the breakdown of cellulose.

Amplitude and phase images were captured to show the changes in secondary cell walls and thickened vascular bundle cell surfaces of native, OAFEX-treated, and enzymatically digested SB (Figure  3a-c). The native SB showed a fibrous network of cellulose, cross-linked lignin, and hemicellulose in the parenchyma of the primary wall (Figure  2a), which is also shown by the AFM image, intact with a uniform surface (Figure  3a). The native SB surface was predominantly hydrophobic (95.40 nm), as confirmed by the darker phase image (Figure 3a). OA specifically disrupts hemicellulose, retaining cellulose and lignin together. The OAFEX-treated SB clearly showed the non-homogeneous phase and globular surface deposition in the amplitude phase (Figure  3b). The AFM tip showed increased affinity toward hydrophilic regions (192 nm) that appear light in color due to a significant change in phase image (Figure  3b). The surface of OA-treated SB is non-uniform, encompassing irregularly shaped hydrophilic deposits, probably due to the exposure of cellulose. Chundawat et al. [28] observed similar patterns in corn stover after pretreatment with ammonium hydroxide.

The presence of globular and irregular shapes (20–95 nm in diameter) can be characteristic of lignin deposits in the cell wall (Figure  3b). This interpretation is in accordance with the SEM analysis, where re-localization of lignin is apparent, linking with cellulose lamellae/ agglomerates (Figure  2b). Lignin re-localization is an important feature that may lead to enhanced enzymatic hydrolysis. OA markedly disrupts hemicellulose and simultaneously re-localizes lignin moieties, aiding the increased exposure of cellulose to cellulases [29,30].

The higher-resolution imaging of AFM reveals maximum non-uniformity in the cellulose lamellae, which can be interpreted as the complete disruption of cellulose aggregates into glucose (Figure  3c). The AFM tip showed high affinity toward hydrophilic areas (192 nm) appearing as changes in phase, and the light color shows the effective accessibility of cellulose to cellulases. Igarashi et al. [31] reported that the real-time visualization of cellulase from *Trichoderma reesei* cellobiohydrolase I action on crystalline cellulose resulted in marked cellulase affinity toward cellulose.

### X-ray diffraction (XRD)

**Figure 4:**
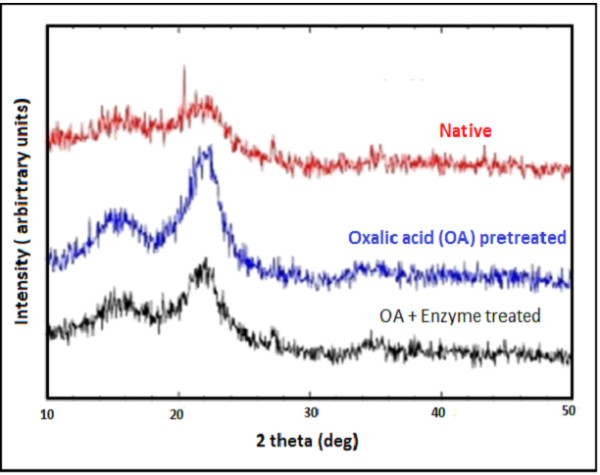
**X-ray diffraction pattern of native, oxalic acid pretreated and enzyme hydrolysed SB.** Crystallinity Index (CrI) was found to be increased in OA pretreated SB and enzymatic hydrolysed SB showing the effect of OAFEX and enzymatic degradation.

XRD analysis revealed the increasing order of crystallinity index (CrI) in OAFEX-treated bagasse (52.56%) and enzyme-digested bagasse (55.65%) compared to native bagasse (45.61%). Figure  4 shows the XRD spectra of native, OAFEX-treated, and enzyme-digested bagasse. In general, after pretreatment of SB, CrI increases with the decrease of amorphous regions in the substrate along with other constituents [6]. The CrI of OAFEX-treated bagasse increases mainly due to removal of hemicellulose in conjunction with re-localisation of lignin and partial disruption of cellulose. Rezende et al. [8] reported that the CrI of consequentially acid–base-pretreated SB increased with a parallel increase in the amount of cellulose in the substrate. Velmurugan and Muthukumar [7] observed a high CrI in sono-assisted alkali-pretreated SB (66%) compared with natural bagasse (50%). The increase in CrI of OAFEX-treated bagasse and enzyme-digested bagasse was due to the depolymerization of hemicellulose and cellulose into their monomeric constituents. Cellulase enzyme cocktails break down the amorphous cellulose, thus increasing the CrI over OAFEX-treated bagasse. However, cellulose crystallinity is not considered to be a principal factor that determines biomass recalcitrance [8].

### Raman spectroscopy

**Figure 5:**
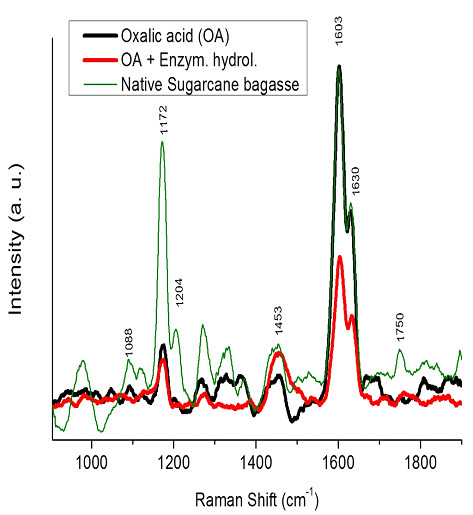
**Raman spectra of native sugarcane bagasse (green online), pre-treated bagasse with oxalic acid (black online) and oxalic acid/enzymatic hydrolysis pre-treated bagasse (red line).** A gradual reduction in band intensities was recorded in OAFEX and enzymatic hydrolysis revealing the displacement in hemicellulose backbone and enzymatic cleavage of β–1–4–glycosidic linkage in cellulose.

Raman spectroscopic analysis revealed a gradual reduction in the band intensity (< 1500 cm^–1^) in OAFEX-treated and enzymatically hydrolyzed SB compared to the native bagasse (Figure  5). The molecular disarrangement and displacement in the hemicellulosic backbone during pretreatment followed by enzymatic cleavage of the β–1–4–glycosidic linkage of cellulose may have caused this gradual reduction in overall band intensities. Previous studies that performed Raman spectroscopic analysis of cellulose and hemicellulose showed that the changes in spectra were dominated by contributions from cellulose at intensities below 1500 cm^–1^[32]. The ratio of the bands at 1172 cm^–1^ and 1204 cm^–1^ reflects the orientation of the cellulose relative to the electric vector (polarization) of the laser [33].

In Raman analysis, the electric field vector has a component along the cellulose axis causing the intensification of the band at 1172 cm^–1^, and considered to obtain the spectral analysis of native, OAFEX-treated, and enzyme-digested SB. Figure  5 shows cellulose/ hemicellulose peaks at 1088 and 1371 cm^–1^. It is evident here that OA pretreatment and enzymatic hydrolysis act on hemicellulose and cellulose respectively. In addition, the main signature of lignin in all three samples is strong band lines at 1603 and 1630 cm^–1^ due to stretching of the asymmetric aryl ring in lignin [33,34]. This may be due to the re-localization of lignin moieties during OA pretreatment of SB at a high temperature. Ooi et al. [34] reported that Raman spectra of native and NaOH-treated Kenaf fibers showed a reduction in intensity of lignin bands in the region around 1750 cm^–1^ assigned to acetyl groups with the carbonyl, C=O group. No significant changes were observed in the intensities of lignin bands at 1750 cm^–1^, maybe due to the fact that OA and enzymatic hydrolysis specifically act on hemicellulose and cellulose respectively.

### Fourier transform infrared spectroscopy (FTIR)

**Figure 6:**
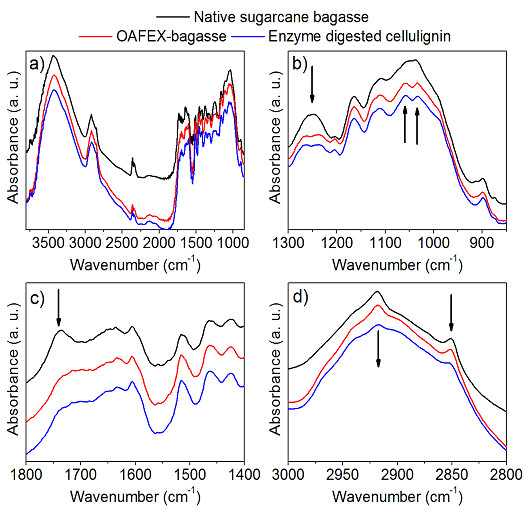
**FTIR spectra of Native sugarcane bagasse (black online), Oxalic acid fiber expansion (OAFEX) (red online) and Enzyme digested cellulignin (blue online). 7b), 7c)** and **7d)** selected regions of Figure 7**a.** The arrows indicate changes observed through the pretreatment. Hemicellulose and cellulose maxima appeared between frequency ranges of 1000–1200 cm^–1^ with maxima at 1037 cm^–1^due to C-O stretching. The absorption around 1463 cm^–1^ refers to CH_2_ and CH_3_ indicating the deformation of lignin while 1606 cm^–1^ is related to C=C stretching of the aromatic ring (lignin) and C=O stretching.

FTIR spectroscopy was carried out to investigate the changes in hemicellulose and cellulose structure during OAFEX treatment and enzymatic hydrolysis. FTIR spectra of the native SB show a band at 900 cm^–1^ representing the β-(1–4) glycosidic linkages of cellulose (Figure  6a-d). The frequency range between 1200–1000 cm^–1^ has a large contribution of hemicellulose and cellulose with maxima at 1037 cm^–1^ due to C-O stretching mode and 1164 cm^–1^ due to the asymmetrical stretching C-O-C [35,36]. The absorption at 1247 cm^–1^ shows due to C–O stretching and is a feature of the hemicelluloses, as well as of lignin [37]. There is divergence about the region at 1316 cm^–1^ (Figure  6b), which can be attributed to vibration of the CH_2_ group of cellulose [35]. Pandey attributes it to the C-O syringyl ring in lignin [38].

The absorption around 1463 cm^–1^ refers to CH_2_ and CH_3_ deformation of lignin, while 1606 cm^–1^ is related to C=C stretching of the aromatic ring (lignin) and C=O stretching (Figure  6c). The absorption around 1515 cm^–1^ is associated with C=C aromatic skeletal vibration [35,38]. The absorption in 1733 cm^–1^ is attributed to a C=O unconjugated stretching of hemicelluloses but also with the contribution of lignin [35,38]. The small peaks at 2850 cm^–1^ and 2918 cm^–1^ come from CH_2_ and CH symmetric and asymmetric stretching respectively (Figure  6d). Both are characteristic of cellulose [36]. The obtained data agree that the range 3800–3000 cm^–1^ comprises bands related to the crystalline structure of cellulose [35]. The region is of great importance and is related to the sum of the valence vibrations of H-bonded OH and intramolecular and intermolecular hydrogen bonds. In the range 1300–1000 cm^–1^, the appearance of two peaks at 1033 cm^–1^ and 1058 cm^–1^ were observed in OAFEX-treated bagasse and enzyme-digested cellulignin spectra respectively (Figure  6b). This indicates penetration of OA in the amorphous region of the biomass and degrading hemicellulose.

Further, the removal of the hemicelluloses is clearly seen by comparing the band around 1247 cm^–1^ with the abrupt decrease of the same band in the OAFEX-treated bagasse (Figure  6b). In the range 1800–1400 cm^–1^, we did not observe changes except around 1733 cm^–1^, which indicates chemical changes in hemicellulose and/or lignin (Figure  6c). Nevertheless, no apparent changes were observed in the lignin-characteristic bands around 1606 cm^–1^, 1515 cm^–1^ and 1463 cm^–1^. Therefore, it is reasonable to state that the hemicellulose was degraded by the action of the OA, which appears to be less stable than lignin. These observations could reflect that the lignin composed of percentages of p-hydroxyphenyl (H), guaiacyl (G), and syringyl (S) units is highly condensed and very resistant to degradation. Further, the 3000–2800 cm^–1^ range shows a decrease of two local maxima mainly in the enzyme-digested cellulignin, indicating the cellulose hydrolysis (Figure  6d).

**Figure 7:**
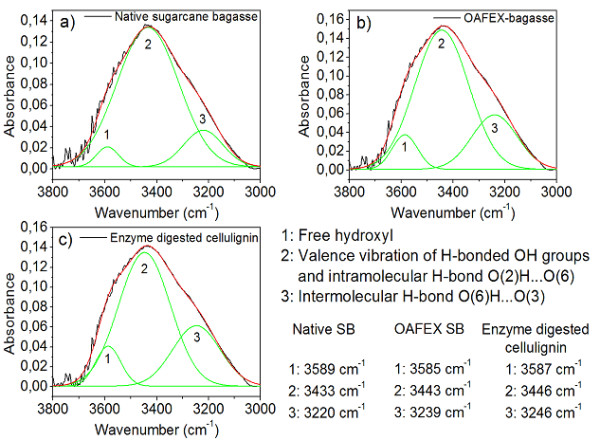
Deconvoluted FTIR spectra of a) Native sugarcane bagasse, b) Oxalic acid fiber expansion (OAFEX) and c) Enzyme digested cellulignin showing: the free hydroxyl (band 1), Valence vibration of H-bonded OH groups and intramolecular H-bond (band 2) and Intermolecular H-bond (band 3).

FTIR spectroscopy, together with the deconvolution technique, provides distinct positions of the bands in this region. Gaussian distribution of the modes in the deconvolution process showed the three bands (Figure  7a-c). The band range from 3310 to 3228 cm^–1^ refers to the intermolecular hydrogen bond O(6)H....O(3) (Figure  7), 3375 to 3335 cm^–1^ belongs to the intramolecular hydrogen bond O(3)H....O(5), and 3460 to 3410 cm^–1^ is related to the intramolecular hydrogen bond O(2)H....O(6) (Figure  7). The band around 3585 cm^–1^ (band 1 in Figure  7) has been reported as the contribution of free hydroxyl [39].

The deconvolution FTIR spectra reveal three bands of the crystalline structure of cellulose (Figure  7). Band 3 of intermolecular hydrogen bonds has shifted to a higher wave number (3220 cm^–1^; 3239 cm^–1^; 3246 cm^–1^), revealing the depolymerization of crystalline cellulose (Figure  7b). Band 2 of intramolecular hydrogen bonds has also shifted to higher frequencies (3433 cm^–1^; 3443 cm^–1^; 3446 cm^–1^) indicating the formation of intramolecular hydrogen bonds when OA penetrated the crystalline structure of cellulose (Figure 7c). This shift is evidence of an energy change in the internal interactions of cellulose [40,41]. The disruption of the structure of plant vegetal fibers and the removal of hemicellulose and/or lignin from the polymer matrix can also configure a closer relationship between the cellulose chains. Another indication of this behavior is the increase in the width and asymmetry of the curves for the OAFEX-treated bagasse.

### Near infrared spectroscopy (NIR)

**Figure 8:**
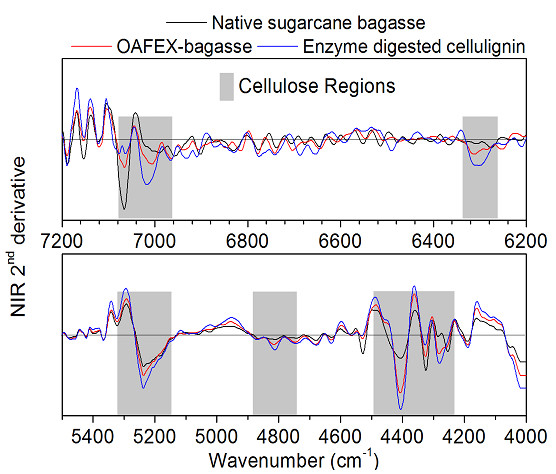
**Second derivative of the NIR spectra (7200 to 4000 cm**^**–1**^**) of native sugarcane bagasse (black online), pre-treated bagasse with oxalic acid (red online) and enzymatic hydrolyzed bagasse (blue online).** In the OAFEX-SB, spectral region 6000 to 5920 cm^–1^ is assigned to the C-H stretching vibration of aromatics due to the re-localization of lignin. Changes in the polysaccharide content after OAFEX and enzyme hydrolysis at regions around 6300, 5208, 4813, 4285, and 4405 cm^–1^.

FT-NIR spectra of native, OAFEX-treated, and enzyme-digested SB ranged from 7200 to 4000 cm^–1^ (Figure  8). The spectral region from 6000 to 5920 cm^–1^ is assigned to the first overtone C-H stretching vibration of aromatics. This could be due to the re-localization of lignin moieties during OA-mediated pretreatment at a high temperature [42,43]. In regard to the hemicellulose structural change, the first overtone C-H stretch around 5800 cm^–1^ is responsible for its variation [42,44]. This reveals that the absorbance of the hemicellulose decreases with diminishing hemicellulosic content in SB. This drastic change in absorbance in hemicellulose is due to the severe pretreatment given to the SB [42].

The stressed regions (grey) around 7000 cm^–1^ correspond to amorphous cellulose (Figure  8). The spectral band around 6290 ±20 cm^–1^ is attributed to crystalline cellulose (CII) and the presence of polysaccharides is shown at 5400 to 4000 cm^–1^[42,44]. Additionally, we observed changes in the polysaccharide content in the regions around 6300, 5208, 4813, 4285, and 4405 cm^–1^. The amorphous region of polysaccharide around 7000 cm^–1^ had a small increase, suggesting the re-localization of lignin units [42].

### Ethanol fermentation

Detoxified hemicellulosic hydrolysate and enzymatic hydrolysates were used for ethanol production using yeasts *C. shehatae* UFMG52.2 and *S. cerevisiae* 174 under submerged culture cultivation. The microorganisms selected for bioconversion of ethanol have been established for the fermentation of xylose and glucose sugars for ethanol production [17,19].

#### Fermentation of acid hydrolysate

**Figure 9:**
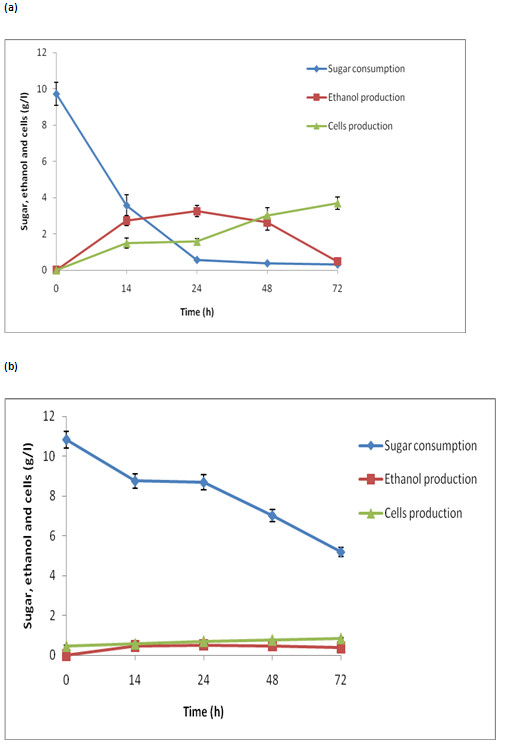
**The time course of growth, sugar utilization and ethanol production using detoxified oxalic acid hydrolysate (fermentable pH of the hydrolysate was adjusted 5.5) by (a)*C. shehatae *UFMG HM52.2 at 30°C (b) *S. cerevisiae *at 30°C.** The values are mean of three replicates. Standard deviation was within 10%.

The fermentation profile of detoxified SB acid hydrolysate from *C. shehatae* UFMG52.2 and *S. cerevisiae* 174 in batch culture is shown in Figure  9a, b. The microorganism *C. shehatae* reached maximum ethanol production (3.20g/l) with a yield of 0.353 g/g and productivity of 0.133 g/l/h from detoxified hemicellulosic acid hydrolysate after 24 h, and declined afterwards (Figure  9a). The biomass continued to increase even after 24h to the completion of the fermentation cycle (72 h), and yielded 0.385 g/g with productivity of 0.0496 g/l/h (Table  2). The detoxified hemicellulose hydrolysate did not show satisfactory ethanol production (Figure  9b) by *S. cerevisiae*, which suggests that the microorganism was unable to utilize the abundance of xylose sugar in the hydrolysate. After 72 h of incubation, a biomass yield of 0.179 g/g and productivity of 0.011 g/l/h were observed (Table  2).

**Table 2:** **Kinetic parameters for ethanol production from detoxified oxalic acid hydrolysate and enzymatic hydrolysates by *Candida shehatae *** **UFMG HM52.2 and *Saccharomyces cerevisiae *** **174**

**Parameters**	**Hemicellulose hydrolysate#**		**Enzyme hydrolysate##**	
	***C. shehatae***	***S. cerevisiae***	***C. shehatae***	***S. cerevisiae***
Initial sugars (g_s_/l)	9.61	10.25	21.03	18.4
Residual sugars (g_s_/l)	0.56	8.14	3.96	0.87
Ethanol (g_p_/l)	3.20	0.52	4.83	6.6
Ethanol yield (g_p_/g_s_)	0.353	0.246	0.282	0.46
Ethanol productivity (g_p_/l/h)	0.133	0.021	0.201	0.47
Biomass (g_x_/l)	3.57	0.85	6.32	4.03
Biomass yield (g_x_/g_s_)	0.385	0.179	0.302	0.22
Biomass productivity (g_x_/l/h)	0.0496	0.011	0.0877	0.055

#During the fermentation of hemicellulosic hydrolysate by *C. shehatae* UFMG HM52.2 and *S. cerevisiae* 174, maximum ethanol was produced after 24 hrs, so ethanol productivity was calculated in both the cases considering 24 hrs. After 24 hrs of incubation, a concomitant downfall in ethanol production was observed with a regular increase in biomass. However, to calculate biomass productivities, incubation time (72 hrs) was considered. ## During the fermentation of enzyme hydrolysate by *C. shehatae* UFMG HM52.2 and *S. cerevisiae* 174, maximum ethanol was produced after 24 hrs and 14 hrs respectively. Therefore ethanol productivities were calculated considering these incubation times. However biomass was found consistently increasing until the completion of fermentation (72 hrs), so biomass productivities were calculated considering 72 hrs of incubation. The values are mean of three replicates. Standard deviation was within 10%.

### Fermentation of enzyme hydrolysate

**Figure 10:**
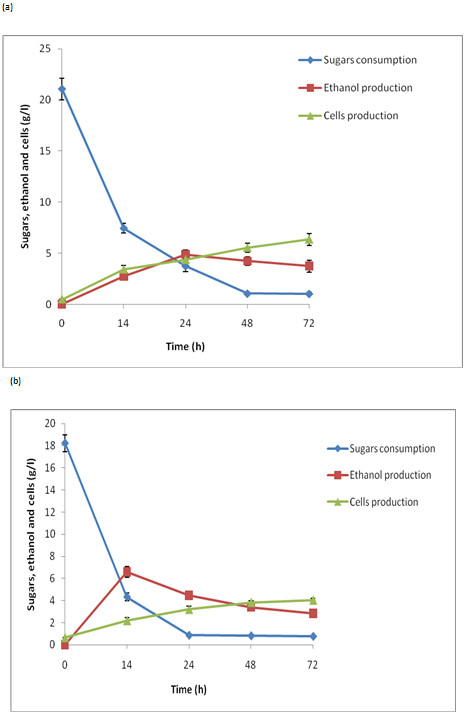
**The time course of growth, sugar utilization and ethanol production using enzyme hydrolysate (fermentable pH of the hydrolysate was adjusted 5.5) by (a) *C. shehatae *UFMG HM52.2 at 30°C (b) *S. cerevisiae *at 30°C.** The values are mean of three replicates. Standard deviation was within 10%.

When the enzymatic hydrolysate was fermented with *C. shehatae* UFMG52.2, maximum ethanol production (4.83 g/l) was found with a yield of 0.282 g/g and productivity of 0.201 g/l/h after 24 h (Figure  10a; Table  2). However, regular growth of microorganisms was observed until the sugar was consumed after 72 h. The maximum biomass production (6.32 g/l) was obtained with a yield of 0.302 g/g and productivity of 0.877 g/l/h. Fermentation of enzymatic hydrolysate with *S. cerevisiae* showed maximum ethanol production (6.6 g/l) after 24 h, with a yield of 0.46 g/g and productivity of 0.47 g/l/h after 14 h of incubation (Figure  10b). The growth in biomass remained unchanged until 72 h of incubation (Figure  10b; Table  2).

*C. shehatae* showed a greater ethanol yield (0.353 g/g) from the acid hydrolysate than from the enzymatic hydrolysate (0.282 g/g), indicating a microbial preference for xylose over glucose as a source of carbon in the fermentation reaction. However, *C. shehatae* is sensitive to fermentation inhibitors present in the hydrolysate. Sreenath et al. [45] showed ethanol production of 5 g/l with a yield of 0.25 g/g by *C. shehatae* FPL 702 from alfalfa hydrolysate. The low ethanol yield of this yeast was probably due to inhibition by pectic acid, organic acids, and hemicellulose-derived inhibitors present in the sugar solution [45]. The hemicellulosic hydrolysates contain mainly xylose, while enzymatic hydrolysates contain only glucose. When *S. cerevisiae* was grown on hemicellulosic hydrolysate, abysmal ethanol production and growth were recorded (Table 2). *S. cerevisiae* does not use xylose present in the hemicellulosic hydrolysate, but rather relies upon glucose, which could be why the poor ethanol production was observed (Figure  9b). Our earlier studies showed that *S. spontaneum* acid hydrolysate after overliming fermented with *S. cerevisiae* VS_3_ in batch conditions produced 1.40±0.07 g/l ethanol with 0.20±0.016 g/g yield after 36 h of fermentation [24].

*C. shehatae* showed a similar growth pattern in both the hydrolysates. In both cases, more than 80% of the sugars were utilized within 24 h of incubation promoting faster growth of microorganism. Similar patterns of biomass growth were observed by Abbi et al. [46], and Chandel et al. [17] reported a regular increase in biomass of *C. shehatae* NCIM 3501 after the exhaustion of xylose in 24 h with the utilization of ethanol as a carbon source for the metabolic growth. Sánchez et al. [47] observed ethanol production (4.5 g/L) from *C. shehatae* CBS4410 utilizing *Paja brava* acid hydrolysate (19.8 g/L xylose, 2.5 g/L glucose) obtained under hydrolysis conditions (180°C, 5 min, 0.5% w/w H_2_SO_4_). Sreenath and Jeffries [48] reported that *C. shehatae* FPL-Y-049 utilized all sugars present in wood hemicellulose hydrolysate except arabinose and produced 34 g/L of ethanol with a yield of 0.46 g/g.

The fermentation of enzymatic hydrolysate (18.4 g/l glucose) by *S. cerevisiae* produced maximum ethanol (6.6 g/L, yield 0.46 g/g) after 14 h of incubation under conditions similar to those employed for hemicellulose hydrolysate fermentation (Figure  10b). It is interesting to note that both yeast strains isolated from Brazilian biodiversity presented a higher growth rate with a shorter lag phase and a prolonged exponential phase. The current growth pattern of both microorganisms supports maximal conversion of sugars into ethanol-an impressive trait for achieving higher ethanol productivity. These characteristics could be beneficial in biorefineries by saving time and reducing the cost of operation and energy.

The maximum ethanol productivity (0.47 g/l/h) was obtained from *S. cerevisiae*-mediated fermentation of OAFEX-treated SB enzymatic hydrolysate, another impressive feature of the *S. cerevisiae* strain used in this study (Table  2). Previously, a natural isolate from spent sulphite liquor, *S. cerevisiae* ATCC 96581, showed maximum ethanol production (7.4 g/l, yield 0.28 g/g and productivity 0.37 g/l/h) from detoxified SB hydrolysate containing 26.0 g/l total sugars [19]. Unlike acid hydrolysates, enzymatic hydrolysate lacks fermentative inhibitors, which eliminates the detoxification step. During the fermentation of enzymatic hydrolysate, *S. cerevisiae* showed less biomass production (4.03 g/l, yield 0.22 g/g) compared to *C. shehatae* (6.32 g/l, yield 0.302 g/g) after 72 h of incubation, suggesting that *C. shehatae* prefers to metabolize a carbon source for ethanol production rather than produce cellular buildup. However, the characteristic of selecting ethanol as a carbon source after the exhaustion of sugars was also observed in *S. cerevisiae* 174.

## Conclusion

Plant cell walls are complex, rigid, and recalcitrant in nature. Pretreatment is an inevitable process to break down this carbohydrate skeleton and increase the accessibility of cellulose. Our studies indicated that OA-mediated hemicellulose degradation of SB is an effective pretreatment strategy that ameliorates the enzymatic hydrolysis of the cellulosic fraction into glucose. The microscopic and spectroscopic techniques used in this work warranted in-depth structural investigation of chemical changes at the molecular level during pretreatment and enzymatic digestion. OAFEX pretreatment significantly removed hemicellulose, causing lignin re-localization, which eventually showed efficient enzymatic action toward the depolymerization of cellulose into glucose (66.51% hydrolytic efficiency). Detoxified hemicellulosic hydrolysate, when fermented by *C. shehatae* UFMG52.2 and *S. cerevisiae* 174, showed ethanol production of 3.20 g/L (yield 0.353 g/g) and 0.52 g/L (yield 0.246 g/g) respectively. Enzymatic hydrolysate after fermentation with *C. shehatae* UFMG52.2 and *S. cerevisiae* 174 produced 4.83 g/L (yield 0.282 g/g) and 6.6 g/L (yield 0.46 g/g) respectively. Both microorganisms revealed high substrate consumption including energy and time savings that could have a major impact in biorefineries.

## Material and methods

### Preparation of raw substrate

The raw substrate, sugarcane bagasse, was acquired from Usina Vale do Rosário (Morro Agudo, S.P, Brazil). In preliminary processing, the SB was air-dried, and knife milled (Marconi Equipamentos, Model No. MA 680, Piracicaba-S.P, Brazil) to pass through with 20-mesh sieve. The finely milled bagasse was washed under running tap water to remove the dust, and dried at 45°C for further experiments.

### Oxalic acid fiber expansion pretreatment of sugarcane bagasse

The pretreatment of the SB with dilute oxalic acid (OA) (3.5% w.v^–1^) was carried out at 160°C for 20 min as described by Scordia et al. [12]. Briefly, SB (10 g d.wt.) and 100 ml (3.5% w.v^–1^) aqueous OA solution (10% total solids) were loaded into a 200 ml stainless-steel container (19x9.7 cm), tightly sealed and immersed in an oil bath provided with electrical heating at 160°C. The container remains resident for 5 min to reach the temperature of 160°C. The hydrolysis was stopped through immersing the container into running water in due course.

After hydrolysis, the hemicellulosic hydrolysate was quantitatively separated by vacuum filtration from the pretreated solids, hereinafter referred to as cellulignin. The cellulignin was thoroughly washed with deionized water and dried in oven at 45°C for 72 h, and subsequently used for enzymatic hydrolysis.

### Detoxification of oxalic acid hydrolysate

OA hydrolysate was detoxified using overliming with the addition of dried calcium oxide under constant stirring until the pH reached 10.5 ± 0.05. The fermentation inhibitors were allowed to precipitate for additional 1 h by stirring. The slurry was then subjected to vacuum filtration using Whatman filter paper #1 to remove the precipitates. The pH of clear filtrate was adjusted to 6.00 ±0.05 with 6 N H_2_SO_4_ and again vacuum filtered to remove traces of salt precipitates.

### Enzymatic hydrolysis

OAFEX-bagasse was enzymatically hydrolysed to depolymerise carbohydrates into simpler sugars. The OAFEX-bagasse (2 g d.wt) was pre-incubated in 40 ml of sodium citrate buffer (50 mM, pH 4.8) in 150 ml Erlenmeyer flask for 1 h at room temperature. The microbial growth was restricted by adding sodium azide (0.005%) during enzymatic hydrolysis. Soaked OAFEX-bagasse was supplemented with different cellulase loadings i.e. 20 FPU/g of the dry substrate from Celluclast 1.5 L, and 30 IU/g of β-glucosidase from Novozym 188 (Sigma-Aldrich, USA). The reaction mixture was supplemented with non-ionic surfactant (2.5 g/l, Tween–20; polyoxyethylene sorbitan monolaurate). The enzymatic hydrolysis was performed at 50°C at 150 rpm in incubator shaker (Innova 4000 Incubator Shaker, New Brunswick Scientific, Enfield, CT, USA) for 96 hrs. The samples (0.5 ml) were collected periodically (24 h), centrifuged at 5000 rpm at room temperature. The supernatant was analyzed for total reducing sugars using DNS method [49]. The extent of hydrolysis (after OA pretreatment and enzymatic hydrolysis) was calculated as follows:

Hydrolysis (%) = reducing sugar concentration obtained × 100/Total carbohydrate content (TCC) in sugarcane bagasse.

### Fermentation

#### Microorganisms and inoculum preparation

Microorganisms *Candida shehatae* UFMG HM 52.2 and *Saccharomyces cerevisiae* 174 were used for the fermentation of the hemicellulose hydrolysates and enzymatic hydrolysates. The microorganism *C. shehatae* UFMG HM 52.2 was isolated from a rotting-wood sample in an Atlantic Rain Forest site (Bello & Kerida Ecological Reserve) situated in the city of Nova Friburgo, Rio de Janeiro state as described by Cadete et al. [50]. *S. cerevisiae* 174 was isolated from the Atibaia river, São Paulo State, Brazil. Both microorganisms were maintened on YPD plates and stored at 4°C.

*C. shehatae* UFMG HM 52.2 was grown in 250 ml Erlenmeyer flasks containing 50 ml of seed medium (30 g/L of sugars (1:1 xylose and glucose), 20 g/l peptone, and 10 g/l yeast extract) in an orbital incubator shaker at 200 rpm at 30°C. Microorganism *S. cerevisiae* was grown in 250 ml Erlenmeyer flasks containing 50 ml of seed medium (30 g/l glucose, 5 g/l peptone, 3 g/l yeast extract, and 0.25 g/l Di-ammonium hydrogen phosphate) in an orbital incubator shaker at 100 rpm at 30°C for 24 h. The cultured microorganisms (*C. shehatae* and *S. cerevisiae*) were centrifuged and prepared for inoculum corresponding to 0.5 g/l cells (d. wt). The prepared inoculums were aseptically transferred into detoxified hemicellulosic and enzymatic hydrolysates (50 ml) supplemented with medium ingredients.

### Ethanol fermentation

The fermentation medium (50 ml) of microorganism *C. shehatae* UFMG HM 52.2 was composed of the hydrolysates (detoxified and enzymatic) supplemented with (g/l): yeast extract (3.0), malt extract (3.0), and ammonium sulfate (5.0) at pH 5.5 [51]. In another set of fermentation, microorganism *S. cerevisiae* 174 was grown in the hydrolysates (detoxified and enzymatic) supplemented with (g/l): yeast extract (1.0), peptone (1.0), Di-ammonium hydrogen phosphate (1.0), Di-potassium hydrogen phosphate (1.0), magnesium sulfate and manganese sulfate (0.5) at pH 5.5 [24]. The hydrolysates were sterilized at 120 °C for 15 min before the use and the medium components were added aseptically prior to addition of inoculum. Fermentation reactions were setup in an orbital incubator shaker (Innova 4000 Incubator Shaker, New Brunswick Scientific, Enfield, CT, USA) at 200 rpm (*C. shehatae* UFMG HM 52.2), and 100 rpm (*S. cerevisiae* 174) for 72 h. Samples (1.0 ml) were collected periodically to determine the production of ethanol, residual sugars and formation of biomass in fermentation broth.

### Analyses

The chemical composition pulverized sugarcane bagasse (native), OAFEX-SB, EH-SB was determined according to the method of Gouveia et al. [52]. Glucose, xylose, arabinose, and acetic acid, concentrations were determined by HPLC (Waters) using Biorad Aminex HPX–87H column at 45°C equipped with refraction index detector. The mobile phase was constituted with sulfuric acid 0.01 N at 0.6 mL/min flow rate as eluent. Furfural and HMF concentrations were also determined by HPLC equipped with Hewlett-Packard RP18 column and UV–VIS detector (2489) (276 nm) at 25°C. Samples were eluted by acetonitrile/water (1:8) supplemented with 1% acetic acid (volume basis) as the eluent at a flow rate of 0.8 ml/min. Total phenolic compounds in hydrolysates were estimated calorimetrically using Folin–Ciocalteu method [53]. Ethanol production was analyzed by HPLC (Waters) using a refraction index detector (2414) and a Biorad Aminex HPX-87H column at 45°C. The growth in biomass *C. shehatae* UFMG 52.2 and *S. cerevisiae* 174 was determined at 600nm using spectrophotometer (Beckman DU640B, USA). The measured absorbance was correlated with the cell concentrations (g/l) following the calibration equation:

Y=2.0029x + 0.0056 (for *C. shehatae* UFMG 52.2)

Y=1.0804x + 0.006 (for *S. cerevisiae* 174)

Pretreatment, enzymatic hydrolysis and fermentation experiments were carried out in triplicates. The values are mean of three replicates.

### Multi-scale visual analysis

#### Scanning electron microscope (SEM)

The SEM analysis of native, OAFEX and enzymatically hydrolysed SB was performed as described by Kristensen et al. [29]. Briefly, native, OAFEX-pretreated and enzymatically hydrolysed SB distributed on a 12 mm glass cover slip coated with poly-L-lysine (Sigma Diagnostics, S.P. Brazil). The dried sections were mounted on aluminum stubs, sputter-coated (JEOL JFC–1600) with a gold layer, and used for scanning. The prepared samples were scanned and imaged using Hitachi S520 scanning electron microscope (Hitachi, Tokyo, Japan).

### Atomic force microscopy (AFM)

The AFM analysis of native, OA pretreated and enzymatically hydrolysed SB was performed as described by Kristensen et al. [29]. All AFM measurements were made with a multi-mode scanning probe microscope with a Nanoscope IIIa controller (Shimadzu SPM-9600 Deluxe, Japan). The images were acquired in tapping mode with etched silicon probe (Nanoworld Point Probe NCHR–10, 320 kHz, 42 N/m). An auto-tuning resonance frequency range of approximately 286–305 kHz with a scan rate of 0.5 Hz and sweep range of 10 kHz was used. The drive amplitude and amplitude set-point were adjusted during measurements to minimize scanning artifacts. Height, amplitude and phase images were captured simultaneously. Scan size varied from 500 nm to 5.0 μm, usually 1 μm.

Samples were fixed on metal discs with double-sided adhesive tape, and the images were measured in air. Images were collected from a minimum of 10 different fibers for each treatment with representative images displayed in the present work. The external vibration noise was eliminated using active vibration-damping table. AFM images were recorded (512 dpi), analyzed and processed (illumination and plane fitting) by the accompanying software.

### X-ray diffraction (XRD)

The crystalline nature of native, OA-treated and enzyme digested SB was analyzed by using a Scifert Isodebye Flex 3003 X-ray diffractometer (Germany). The crystallinity was analysed by adjusting the diffractometer set at 40 KV, 30 mA; radiation was Cu Kα (λ = 1.54 Å). Samples were scanned over the range of 100 <2θ <500 with a step size of 0.05° and the crystallinity index (CrI) were determined using the empirical method described by Segal et al. [54]:

$$\mathrm{CrI}=\frac{I\;\mathrm{crytalline}-I\;\mathrm{amorphous}}{I\;\mathrm{crystalline}}\;X\;100\%$$

Where, I crystalline = intensity at 21°C and I amorphous = intensity at 18.8°C.

### Raman spectroscopy

The Raman spectra of native, OA pretreated and enzyme digested SB were recorded via microRaman (T64000 Horiba Jobin Ivon, France) using the 488nm line of an argon laser, 1800 g/mm grating with a 50x objective. The samples were passed through the laser power of ~2 mW, and the scattered light were detected by a CCD system cooled with liquid nitrogen at the temperature of −130°C. To maximize the Raman signals, we have used the fact that the Raman intensity of the cellulose is dependent of the laser light polarization. Cellulose mediated polarization of laser light is dependent on the position of sample at the microscopic stage. The spectra are scaled to the cellulose peak at 1172 cm^–1^, baseline was subtracted and smoothed with adjacent-averaging with 30 points.

### Fourier transform infrared spectroscopy (FTIR)

FTIR spectroscopic analysis was performed to detect the changes in functional groups after OA pretreated and enzyme digested SB in comparison to native SB. Samples were put to mill in agate cups for 1.5 hrs with 400 rpm (RETSCH® PM 400) followed by passing through with an abronzinox sieve of 100 mesh size with an aperture of 150 μm. The pellets were prepared by mixing of 300 mg of spectroscopic grade KBr with 3 mg of sample in an agate mortar. Each sample was then submitted to 10 tons for 3 min in a hydraulic press (SPECAC 25T, ATLASTM). The spectra were collected in a VERTEX 70 spectrometer (Bruker Optics, Germany) and submitted to 4 cm^–1^ of resolution and 72 scans per sample.

### Fourier transform-Near infra-red spectroscopy (FT-NIR)

The FT-NIR spectroscopy of native, OA pretreated and enzyme digested SB samples was performed with a spectrometer FT-NIR MPA (multi-purpose analyser) from Bruker Optics, Germany. The measurements have used to diffuse reflectance which were analysed via an integrating macro sample sphere, the diameter of measured area was 15 mm, 32 scans per sample were performed with a resolution of 4 cm^–1^ covering a range from 13000 to 3500 cm^–1^. Second derivative spectra were calculated with 21 smoothing points after unit vector normalization. All calculations were conducted with OPUS 6.5 version software.

## Competing interests

The authors declare that they have no competing interests.

## Authors’ contributions

AKC planned and performed the biomass pretreatment, enzymatic hydrolysis, ethanol fermentation, as well as the analysis of the results and manuscript writing. AKC also coordinated the overall study. FAFA assisted in biomass characterization, fermentation experiments and helped the manuscript drafting. VA, MJVB and LNR jointly carried out the Raman Spectroscopy, FTIR and FT-NIR analysis and written related text in the manuscript. OVS analyzed all the results and reviewed the manuscript draft. CAR and FCP provided the yeast strains and fermentation methodology. Both analyzed the fermentation results and contributed to the drafting of the text related to fermentation. SSS coordinated the overall study, analysis of results and finalizing the manuscript. All authors suggested modifications to the draft and approved the final manuscript.
